# Treatment Use Among U.S. Adults with a Substance Use Disorder: Associations with Symptom Severity, Problem Self-Perception, Comorbid Mental Illness, and Mental Health Treatment

**DOI:** 10.3390/ijerph22040640

**Published:** 2025-04-18

**Authors:** Namkee G. Choi, C. Nathan Marti

**Affiliations:** Steve Hicks School of Social Work, University of Texas at Austin, 405 W 25th St, Austin, TX 78705, USA; nate.marti@utexas.edu

**Keywords:** substance use treatment, perceived need, symptom severity, problem self-perception, mental illness, self-sufficiency beliefs, stigma

## Abstract

Using data from the 2022 and 2023 National Survey on Drug Use and Health, we examined factors associated with treatment use for substance use disorder (SUD), perceived SUD treatment needs, and reasons for treatment non-use. Of U.S. adults, 18.1% had any past-year SUD (alcohol use disorder [AUD] and/or any drug use disorder [DUD]), 14.4% of those with SUD received SUD treatment in the past year, and 5.5% of those who did not receive treatment had a perceived need for treatment. Treatment use was significantly associated with AUD and DUD severities (aOR = 3.85, 95% CI = 2.82–5.26 for severe AUD; aOR = 2.82, 95% CI = 2.27–3.47 for severe DUD), problem self-perception (aOR = 2.12, 95% CI = 1.74–2.58), and mental health treatment use (aOR = 6.07, 95% CI = 4.73–7.78). Perceived treatment needs among those who did not use treatment were also significantly associated with AUD and DUD severities, problem self-perception, and any mental illness. The most frequently reported reasons for treatment non-use among those with perceived need were self-sufficiency beliefs, lack of readiness to stop using or start treatment, stigma-related concerns, and health insurance/cost problems. The findings underscore the importance of screening SUD and educating about the harms of untreated SUD in increasing motivation and readiness for treatment use among people with SUD.

## 1. Introduction

According to the 2023 National Survey on Drug Use and Health (NSDUH), 17.1% of the USA population age 12 years and older, or 48.5 million people, had a past-year substance use disorder (SUD) per *DSM-5* criteria [1], including 28.9 million who had an alcohol use disorder (AUD) and 27.2 million who had a drug use disorder (DUD) [2]. Of those with a past-year SUD, 14.6%, or 7.1 million people, received substance use treatment in the past year in an inpatient and/or outpatient location, via telehealth, in a prison, jail, or juvenile detention center, or medication-assisted therapy for alcohol use or opioid use [2].

SUDs cause significant harm to individuals through negative physical and mental health effects, functional impairments, and social and legal problems, while also burdening society with increased healthcare costs, crime, lost productivity, and strained social services. Especially given the increased drug overdose death rates (from an age-adjusted rate of 8.2 per 100,000 standard population in 2002 to 32.6 in 2022 and 31.3 in 2023 [3,4]), the high SUD rate and the low treatment use rate are concerning. It is important to identify facilitators and barriers to treatment receipt, as psychosocial and pharmacological treatments can be effective in reducing substance use and improving physical and mental health [5,6,7,8,9,10]. Previous research on treatment receipt barriers identified both personal-level and environmental/structural factors. Personal-level barriers include the lack of readiness to stop using substances, a low-perceived need for treatment, personal and public stigma, concerns about the potentially negative social, and/or occupational effects of the revelation of substance use problems, self-sufficiency beliefs (i.e., they should be able to handle their alcohol or drug use on their own), poor health, and poly-substance use. Environmental/structural barriers include lack of insurance, financial problems, lack of available services, organizational configuration of service providers, and service and residential location [11,12,13,14,15,16,17,18]. Compared to urban areas, non-metropolitan counties or large and small rural areas were associated with decreased odds of receiving treatment, partly due to the lack of treatment programs and providers in less densely populated areas [12].

Research has also shown that co-occurring mental illness and mental health treatment can be used as avenues for substance use treatment [13,19]. Given the high prevalence and harms associated with comorbid mental disorders and SUD, mental health providers are routinely recommended to assess SUD and provide care, and many do provide such services [20]. A study of the characteristics of the USA mental health and substance use service facilities in 2019 found that approximately 60% of mental health service facilities provided substance use treatment [21]. A systematic review also identified some promising evidence for workplace-based universal health promotion interventions, targeted brief interventions, and universal substance use screening; however, lack of engagement, reluctance to seek help amongst male employees, confidentiality concerns, and heavy use were implementation barriers [8]. Other studies found that those who were involved with the criminal justice system (e.g., arrested or on probation, parole, or supervised release) had higher odds of receiving treatment [18,22,23]. A study reported that the proportion of referrals to specialty SUD treatment from the legal system declined between 2015 and 2019; however, referrals from probation/parole and diversionary programs grew over time [24].

Despite extant research on facilitators and barriers to SUD treatment receipt, some research gaps remain. First, while previous epidemiologic studies of SUD treatment receipt [11,16,17] have been helpful, an updated analysis with more recent data is needed to examine whether the findings of these studies still hold in more recent data. A study using the 2017 NSDUH found that addiction severity based on *DSM-5* and probation status were the two most important predictors of treatment receipt, and people with severe opioid use disorder had the highest probability of treatment receipt [25]. A study based on the 2021 NSDUH found that college students who received SUD treatment had more severe SUD symptomology and a greater prevalence of stimulants, opioids, tranquilizers, and poly-SUDs than those who did not receive treatment [26]. Finally, a study based on the 2022 NSDUH data showed that adults with moderate or severe, compared to mild, cannabis use disorders, as well as moderate and severe mental illness, had significantly higher odds of receiving SUD treatment [27]. These studies suggest that SUD symptom severity, along with poly-SUDs and mental illness severity, is a significant factor for treatment receipt. More research is needed to examine the role of SUD symptom severity, together with other behavioral health needs, in treatment receipt and the perceived need for treatment. Second, more research is also needed to examine the role of self-perception of substance use problems in treatment receipt and the perceived need for treatment. Along with SUD symptom severity, problem self-perception is likely an important driver of treatment receipt and the perceived need. Third, previous research on barriers to treatment use was often conducted with those who perceived treatment need but did not use it and focused on their self-reported reasons for not accessing treatment [6,11,13,27]. We also need to examine the differences between those with and without the perceived need with respect to their demographic and clinical characteristics.

In the present study, using the combined 2022 and 2023 NSDUH data, we intended to fill these research gaps by examining the role of SUD symptom severity, problem self-perception, poly-SUDs, mental illness severity, and mental health treatment as contributors to treatment use and perceived treatment need. We applied Andersen’s behavioral model of healthcare use [28,29] as the conceptual framework for the study. Andersen’s model posits that predisposing, enabling, and need factors influence individuals’ health service use. Predisposing factors are individual biological and demographic characteristics and health beliefs, and enabling factors are the resources facilitating access to care. Need factors refer to perceived/evaluated physical and/or behavioral health problem severity and tend to be the strongest predictor of physical [30] and behavioral health service use [31,32].

We posited the following study hypotheses: controlling for predisposing and enabling factors; (H1) among those with any past-year SUD, moderate or severe symptom severity, problem self-perception, dual AUD and DUD, and the severity of mental illness and mental health treatment use as indicators of need would be associated with higher odds of past-year any SUD treatment use; and (H2) among those who did not receive any SUD treatment in the past year, these same need factors would be associated with higher odds of seeking or thinking about treatment (i.e., perceived need) compared to not seeking/thinking about treatment (i.e., no perceived treatment need). The predisposing factors were demographic characteristics, including residential area; enabling factors were education, income, insurance, availability of help with substance use problems at the workplace, referrals from probation/parole programs, and health status (e.g., poor health as a barrier). We also examined self-reported reasons for treatment non-use among those who had perceived need. The study findings add to the knowledge base regarding SUD and SUD treatment prevalence among those with SUD and the correlates of SUD treatment receipt and not seeking treatment.

## 2. Materials and Methods

### 2.1. Data Source

The NSDUH, funded by the USA SAMHSA, is the largest annual survey of substance use, mental health, and behavioral health treatment with a nationally representative sample of the civilian, non-institutionalized population age 12 and older. NSDUH began using the *DSM-5* SUD criteria and reporting symptom severity in 2021. *DSM-5* SUD diagnoses showed greater reliability than *DSM-IV* diagnoses of abuse or dependence [33,34]. For a larger sample, we combined the 2022 and 2023 NSDUH data. The 2021 data were not included in the present study because substance use treatment and mental health treatment questions underwent considerable revision for the 2022 NSDUH [35]. Thus, the 2022 and 2023 NSDUH estimates for these treatments should not be compared with estimates from 2021 or prior years [36]. NSDUH uses a multi-stage, stratified sampling design, with states as the first level of stratification and an independent, multistage area probability sample within each state and the District of Columbia [36]. Because of the ongoing COVID-19 pandemic, the 2022 and 2023 data were collected using both in-person and web-based modes. The overall percentage of interviews that were completed via the web was 40.7% in 2022 and 36.0% in 2023 [2,35].

### 2.2. Participants and Procedures

The 2022 and 2023 NSDUH public use data sets included responses from 59,069 and 56,705, respectively, individuals age 12 and older who completed an in-person or web-based NSDUH survey. In the present study, we focused on 92,233 (47,100 in 2022 and 45,133 in 2023) people age 18 years and older, and then 19,555 (9869 in 2022 and 9686 in 2023) people with past-year AUD and/or DUD. NSDUH’s multi-stage area probability sampling design made it unlikely that any duplication of survey respondents occurred in the pooled two years of survey data. An analysis of these de-identified public-use data was exempt from the authors’ institutional review board review.

### 2.3. Measures

#### 2.3.1. Past-Year Receipt of Substance Use Treatment

Starting from the 2022 NSDUH, people were classified as having received substance use treatment if they received treatment in the past year for the use of alcohol or drugs in an inpatient location (including residential rehabilitation or treatment centers); in an outpatient location; via telehealth; or in a prison, jail, or juvenile detention center (not mutually exclusive). People were also classified as having received substance use treatment if they received medication-assisted treatment (MAT) for their use of alcohol or opioids. While not classified as “substance use treatment”, NSDUH collected data on the receipt of support services from a support group or a peer support specialist or recovery coach, services in an emergency room, or detoxification or withdrawal support services.

#### 2.3.2. Substance Use Treatment-Seeking vs. Not Seeking

We classified those with past-year SUD who did not receive treatment into two groups: (1) those who sought treatment or thought they should receive treatment (but did not use treatment), and (2) those who did not seek treatment or thought that they did not need treatment. The first group provided reasons for not receiving treatment. Hereafter, we refer to these two groups as treatment seekers (with perceived need) and treatment non-seekers (without perceived need).

#### 2.3.3. Past-Year SUD

Respondents were asked SUD questions, per the 11 *DSM-5* criteria, for any alcohol or drugs they used in the 12 months prior to the survey. Any past-year SUD refers to an AUD or a DUD. Drugs included in the DUD assessments were cannabis/marijuana, cocaine (including crack), heroin, hallucinogens, inhalants, methamphetamine, and any use of prescription pain relievers, tranquilizers or sedatives (e.g., benzodiazepines), and stimulants. SUDs were categorized as AUD only, DUD only, or both AUD and DUD.

#### 2.3.4. SUD Severity

For all individual SUDs, the *DSM-5* SUD criteria for severity level classification were used for mild (meeting 2–3 criteria), moderate (meeting 4–5 criteria), and severe (meeting 6+ criteria) disorders. For SUD measures that were aggregated across more than one substance (e.g., any SUD, DUD), mild SUD meant that people had only mild SUDs; moderate SUD meant that people had at least one moderate SUD but did not have severe SUDs; and severe SUD meant that people had a severe SUD for at least one substance [2].

#### 2.3.5. Problem Self-Perception

The NSDUH included a variable on whether or not the respondent had ever perceived an alcohol or drug use problem (no = 0, yes = 1).

#### 2.3.6. Past-Year Mental Illness Severity and Mental Health Treatment Use

Mental illness referred to any mental, behavioral, or emotional disorder in the past year of sufficient duration to meet criteria from the *DSM-IV*, excluding developmental and substance use disorders. Mental illness was further classified as mild, moderate, and serious (i.e., when the mental illness substantially interfered with or limited one or more major life activities) based on statistical prediction models that were developed using clinical interview data from a subset of NSDUH adult respondents aged 18 or older between 2008 and 2012 [2]. Past-year mental health treatment use referred to treatment received in an inpatient or outpatient location, prescription medication, via telehealth, or a prison, jail, or juvenile detention center.

#### 2.3.7. Predisposing Factors

These included age group (18–25, 26–34, 35–49, 50–64, 65+); sex (male vs. female); race/ethnicity (non-Hispanic white, non-Hispanic Black, Hispanic, Asian/Pacific Islander, American Indian/Alaska Native, multi-racial); marital status (married, widowed, divorced/separated, never married); and residential area (large metropolitan area, small metropolitan area, non-metropolitan area).

#### 2.3.8. Enabling Factors

These included education (<high school, some college/associates degree, college degree); income (<poverty, up to 2× poverty, >2× poverty); any health insurance coverage (yes or no); availability of help with substance use problems at the workplace (yes or no); past-year probation/parole status (yes or no); and health status (number [0–10] of chronic medical conditions [asthma, hypertension, diabetes, heart disease, COPD, cirrhosis of the liver, hepatitis, HIV/AIDS, kidney disease, and cancer] that a health professional diagnosed).

### 2.4. Analysis

We used Stata/MP 18’s svy function (College Station, TX, USA) and subpop command in all analyses to account for NSDUH’s multi-stage, stratified sampling estimates to ensure that variance estimates incorporate the full sampling design. For this study’s 2-year pooled data set, adjusted person-level analysis weights were created by dividing the final person-level analysis weights by the number of years of combined data (two in the current study) following NSDUH guidelines for combining sampling weights across multiple survey years. All estimates presented in this study are weighted except for sample sizes. First, we calculated the percentages, with 95% confidence intervals (CI), of past-year SUD, SUD treatment use and non-use, and seeking and not seeking treatment among those who did not receive treatment. Second, we used Pearson’s *χ*^2^ or *t* tests to compare predisposing, enabling, and need factors (1) between SUD treatment users and non-users among those with SUD, and (2) between treatment seekers and non-seekers among treatment non-users. Third, we used two logistic regression models to test the study hypotheses (H1: correlates of SUD treatment use vs. non-use; H2: correlates of treatment-seeking vs. non-seeking). We present logistic regression model results as adjusted odds ratios (aOR) with 95% CI. We used the variance inflation factor (VIF) as a preliminary diagnostic to assess multicollinearity among covariates, applying a cut-off of 2.50 [37] from linear regression models. VIF diagnostics indicated that multicollinearity was not a concern. Significance was set at *p <* 0.05. Finally, we presented the self-reported reasons for treatment non-use among treatment seekers in percentages of treatment seekers.

## 3. Results

### 3.1. Prevalence of Past-Year SUD and Treatment Receipt

**Figure 1:**
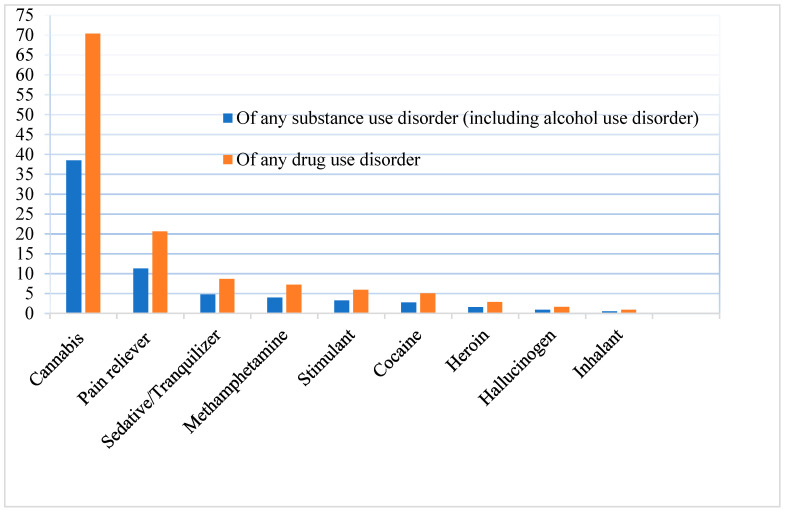
Type of drug use disorders (%).

Table 1 shows that in 2022 and 2023, 18.1% of USA adults (or 46.4 million people), without any significant difference between 2022 and 2023 (F[1, 50] = 0.33, *p* = 0.566), had any past-year SUD. Of those with any SUD, 16.1% had both AUD and DUD, 45.3% had AUD only, and 38.6% had DUD only—i.e., 61.4% had AUD and 54.7% had DUD—again without any significant difference between 2022 and 2023 (F[1.95, 97.50] = 1.23, *p* = 0.296). Figure 1 shows specific DUDs, with cannabis use disorder being the most prevalent, followed by prescription opioid/pain reliever disorder.

Of those with any SUD, 14.4% (or 6.7 million people) received any SUD treatment in the past year, without any difference between 2022 and 2023 (F[1, 50] = 0.220, *p* = 0.641). Of those who did not receive any treatment, 5.5% reported that they sought treatment or thought to receive treatment, and 94.5% did not. Additional analysis of those who used any past-year treatment showed that 33.2% received it in an inpatient setting, 74.7% in an outpatient setting, 37.9% received telephone/video-based treatment, and 33.0% received MAT. Of those who received any of these SUD treatments, 16.8% reported also having had any emergency department visits for substance use problems, 15.3% had detoxification services, 20.0% had peer support specialist services, and 31.8% attended support groups. Please note that these service uses were not mutually exclusive. Of those who did not receive any SUD treatments, only between 0.1% (detoxification) and 1.9% (support group) reported the use of these other services.

### 3.2. Characteristics of SUD Treatment Users and Non-Users

Table 2 shows that, compared to treatment non-users, treatment users were older, included higher proportions of divorced/separated individuals, those with ≥high school degree and incomes below poverty, and those on parole/probation, but they included lower proportions of non-metropolitan area residents and those with workplace-based help with substance use problems, and had more chronic medical conditions. Treatment users also included higher proportions of those with DUD, with or without AUD, those with severe AUD and DUD symptoms, those with problem self-perception, those with any and severe mental illness, and those who received mental health treatment in the past year. Additional analysis showed that the higher the SUD symptom severity, the higher the rates of problem self-perception. Specifically, 23.2%, 42.8%, and 67.3% of those with mild, moderate, and severe AUD symptom severities, respectively, had self-perceptions (F[2.76, 137.88] = 167.46, *p* < 0.001); 26.1%, 34.6%, and 60.0% of those with mild, moderate, and severe DUD symptom severities, respectively, had self-perceptions (F[2.79, 139.30] = 98.98, *p* < 0.001).

### 3.3. Treatment Non-Users: Comparison Between Treatment Seekers and Non-Seekers

The last three columns of Table 2 show that, compared to treatment non-seekers, treatment seekers included higher proportions of individuals aged 26–34 years, females, and those with a college degree, but a lower proportion of those with any health insurance. Treatment seekers also included higher proportions of those with DUD, with or without AUD, those with severe AUD and DUD symptoms, those with problem self-perception, those with any and severe mental illness, and those who received mental health treatment in the past year.

### 3.4. Associations of Treatment Use with SUD Symptom Severity, Problem Self-Perception, Comorbid Mental Illness, and Mental Health Treatment

The first column of Table 3 shows that controlling for predisposing and enabling factors, AUD and DUD symptom severities were significantly associated with the odds of treatment use, with severe symptoms having the highest odds (AUD: aOR = 3.85, 95% CI = 2.82–5.26; DUD: aOR = 2.82, 95% CI = 2.27–3.47), followed by moderate symptoms (AUD: aOR = 1.63, 95% CI = 1.18–2.25; DUD: aOR = 1.54, 95% CI = 1.20–1.97). Problem self-perception was also associated with higher odds of treatment receipt (aOR = 2.12, 95% CI = 1.74–2.58). Compared to AUD only, DUD only was also significantly associated with higher odds (aOR = 1.87, 95% CI = 1.39–2.52), but dual AUD and DUD was not. Those who received mental health treatment were also six times more likely to have received SUD treatment (aOR = 6.07, 95% CI = 4.73–7.78), but the severity of mental illness was not a significant factor.

Of predisposing factors, older ages compared to the 18–25 years, male sex, and non-married state were associated with higher odds of treatment receipt. Of enabling factors, any health insurance coverage and parole/probation status were associated with higher odds, but having some college education or a college degree was associated with lower odds.

### 3.5. Correlates of Treatment-Seeking Among Treatment Non-Users

The second column of Table 3 shows that AUD severity (aOR = 1.95, 95% CI = 1.21–3.11 for moderate symptoms; aOR = 7.07, 95% CI = 4.72–10.57 for severe symptoms), DUD severity (aOR = 1.79, 95% CI = 1.09–2.96 for moderate symptoms; aOR = 6.85, 95% CI = 4.30–10.91 for severe symptoms), problem self-perception (aOR = 6.47, 95% CI = 4.59–9.10), and mild, moderate, and severe (aOR = 2.14, 95% CI = 1.39–3.29) mental illness, compared to not having any past-year mental illness, were associated with increased odds of treatment-seeking or thinking to receive treatment (but did not use it) compared to treatment non-seeking. The only other significant correlate was age: compared to the 18–25 age group, the 26–34 and 50–64 age groups had higher odds of treatment-seeking but not receiving it.

### 3.6. Personal and Environmental/Structural Reasons for Treatment Non-Use Among Treatment Seekers

Table 4 shows that of those who sought treatment or thought to receive treatment but did not receive it, the most common reason for not receiving treatment was self-sufficiency beliefs. More than three-quarters reported not using treatment because they thought they could handle the problem independently. Over 60% and 50% also reported that they were not ready to start treatment and to stop/quit using substances. About 48% thought the treatment cost was too high; 45% did not know where to go; 44% worried about what people would think/say; and 42% reported not having had enough time for treatment. Over one-third each reported no insurance, insufficient insurance coverage, and concerns about confidentiality. One-third also reported not finding a preferred provider; 27% thought that treatment would not help; and a little less than a quarter worried about being coerced. Less than one-fifth, respectively, thought that no one would care if they became better and family/friends/religion would not like it. Less than 15% reported no opening with a preferred provider.

## 4. Discussion

This study examined the role of SUD symptom severity, problem self-perception, mental illness severity, and mental health treatment use in SUD treatment receipt and perceived treatment need among USA adults with any SUD. Our findings show that 18.1% of the adult population in 2022–2023 had past-year AUD or DUD, and only 14.4% of them received past-year treatment. Our findings also show that, of those who did not receive any treatment, only 5.5% sought treatment or thought about receiving treatment, indicating that treatment non-use was largely attributable to the lack of perceived need for treatment.

### 4.1. Interpretation of the Findings

As hypothesized, greater SUD symptom severity was a significant factor for treatment use and perceived treatment need, indicating that the experience of multiple problems from their substance use impelled treatment use and engendered perceived treatment need. Severe symptom severity for both AUD and DUD had greater odds than moderate severity for treatment use, suggesting gradient effects. Although further research is necessary to identify the most frequently endorsed *DSM-5* criteria for moderate and severe AUD and/or DUD, any problems associated with loss of control, failure to fulfill role obligations, relationship/social conflicts, and physical and psychological toll can have devastating effects on the individual, family, and societal well-being [38,39]. More problems would result in more negative consequences, necessitating treatment. While legal problems are not part of the *DSM-5* criteria [40], epidemiologic data showed that adults reporting current alcohol-related legal problems were 22 times more likely to have a current AUD diagnosis, and adults with lifetime drug-related legal problems were 3–5 times more likely to have a current and lifetime DUD diagnosis [41].

Our findings also show the significance of problem self-perception as a driver of treatment use and perceived treatment need. The relationship between the greater SUD severity and the higher rates of problem self-perception again suggests that multiple problems from their substance use prompted admission of the problems. Drug addiction has been associated with impaired insight, drug-biased attention, lack of self-awareness, and negative outcome insensitivity, possibly due to aberrant functioning of self-referential brain circuitry [42,43]. However, our findings show that SUD symptom severity contributes to an increased likelihood of self-awareness of SUD-related problems and treatment use. Among treatment non-users, controlling for a variety of predisposing and enabling factors, those who had problem self-perception were six times more likely to have sought treatment or thought to receive treatment than those who did not have the self-perception. Little previous research has been conducted on the role of problem self-perception in treatment use; however, a change in substance self-concept (e.g., viewing oneself as a drinker or smoker) has been found to be associated with substance use patterns, outcomes, and recovery from substance misuse [44].

We hypothesized that those with dual AUD and DUD, compared to those with either individual disorder, would have higher odds of treatment use and higher perceived need for treatment. However, the results were that, compared to AUD alone, DUD alone had higher odds for treatment use, and that SUD type was not a significant factor for perceived need. A ranking of the harm of psychoactive drugs, including prescription analgesics, found crack, methamphetamine, heroin, alcohol, and cocaine to be the top five harmful substances to the self and others [45]. The age-adjusted alcohol-induced death rate (13.2 per 100,000 standard population in 2020) is lower than drug-overdose death rates; however, the rates for males ages 55–64 (59 per 100,000) and 65–74 (43.4 per 100,000) show the need for AUD treatment for older males with AUD [46].

A significant association between SUD treatment use and mental health treatment use was expected, as mental health treatment in both general medical and specialty treatment settings likely involved co-occurring SUD and mental health problems, as discussed earlier. Among SUD treatment non-users, while the mental health treatment receipt was not a significant factor, having any mental illness was associated with greater odds of perceived need, which indicates the need for both SUD and mental health treatment. Given that 30.8% of SUD treatment non-users received mental health treatment, it can be a good segue leading to SUD treatment.

Of predisposing and enabling factors, race/ethnicity was not significantly associated with treatment use and perceived need, but age was a significant factor. Compared to the 18–25 age group, the 26–34 and 50–64 age groups were more likely to have perceived need but did not receive treatment. The finding that those with some college education and college graduates had lower odds of treatment receipt, compared to those with ≥high school degrees, is consistent with the findings of previous studies [13,27] and suggests lower rates of perceived need for treatment among them [47]. Those with higher income also had a lower likelihood of treatment use and perceived need, but health insurance coverage and parole/probation status were facilitators. As discussed, the significance of parole/probation status suggests the criminal justice/legal system mandate for treatment. Workplace-based assistance was not a significant factor. We speculate that concerns about negative effects on jobs, as shown in the reported reasons for treatment non-use, may lead to not utilizing such a program.

Finally, of the self-reported reasons for treatment non-use despite the perceived need, personal-level barriers, especially self-sufficiency beliefs, lack of readiness, and stigma-related concerns, were more frequently endorsed than structural/environmental barriers, although cost/limited insurance remained a significant barrier. Given that the NSDUH definitions of SUD treatment have changed since 2022, direct comparison is not warranted; however, self-sufficiency beliefs (76%) drastically increased from 6% in the 2008–2012 NSDUH [11]. Self-sufficiency reasons may stem from the harm-reduction and self-management strategies that some people with chronic substance use engage in [48,49]. With the proliferation of digital and artificial intelligence-driven self-help apps in recent years, we also speculate that people may be more likely to think they can treat SUD without help from health professionals. Systematic reviews showed that app-based interventions alone might have uncertain outcomes, but augmenting evidence-based treatment with them may be beneficial [50,51].

Stigma-related concerns in 2022–2023 appeared to have doubled from 2008 to 2012, which may reflect the high level of public stigma regarding SUD and the sense of shame felt by those with SUD. A systematic review found that compared to substance-unrelated mental disorders, the public desire for social distance was consistently higher for people with AUD/other SUD, as they were perceived as being more dangerous and responsible for their condition [52]. The personal and moral failure view of people with substance use problems is often internalized among those with SUD and can significantly interfere with a willingness to seek treatment [53]. Concerns about negative opinions and discrimination from other people, including health professionals [54], can exacerbate psychological distress and shame and lead to the desire to take treatment in their own hands rather than seeking professional help. We speculate that stigma-related concerns may have increased over the past decades with heightened psychological distress and shame amid the societal crisis of rapidly increasing drug overdose deaths.

### 4.2. Limitations

The study limitations are as follows: First, the validity of respondents’ self-reported SUD and mental health treatment use was not checked. The potential recall and social desirability biases may have affected the outcomes. Second, the time frame for problem self-perception was not limited to the past year, although recognition of problems at any point in time could have influenced past-year treatment use. Third, cross-sectional data could only show correlation, not a causal relationship. Fourth, treatment duration and effectiveness, which likely varied widely, were not examined. Fifth, an analysis of different types of treatment, such as MAT, is also needed. Despite these limitations, the findings significantly add to the existing knowledge base regarding SUD treatment facilitators and barriers.

### 4.3. Clinical Practice and Policy Recommendations

Despite these limitations, our findings underscore a critical treatment gap in addressing SUDs in the USA and the urgent need for multifaceted clinical and policy responses to increase SUD treatment receipt. Given that most treatment non-users reported not even thinking about seeking treatment, clinical strategies must focus on alcohol/drug harm education and other proactive engagement strategies, including universal SUD screening in primary care and mental health settings and motivational interventions that address patient ambivalence, stigma, beliefs around self-sufficiency beliefs, and readiness for change. Policy efforts should prioritize expanding insurance coverage, reducing out-of-pocket costs, and integrating public awareness and stigma-reduction campaigns to normalize help-seeking. It is important that community-level outreach programs with age-appropriate and culturally responsive messaging reach individuals who are not even contemplating treatment. Together, these approaches can lower personal/psychological and environmental/structural barriers, ultimately closing the treatment gap and improving health outcomes for individuals with SUD.

## 5. Conclusions

Nearly one fifth of the USA adult population in 2022–2023 had past-year AUD or DUD, but less than one in six of them received past-year treatment. An absolute majority of the treatment non-users did not perceive that they needed treatment. Among those with perceived treatment needs, self-sufficiency beliefs, lack of readiness, stigma-related concerns, and treatment cost were barriers to treatment use. On the other hand, SUD symptom severity and problem self-perception were significant factors of treatment use. The significance of symptom severity and problem self-perception points to the importance of screening SUD and educating about the harms of untreated SUD in increasing motivation and readiness for treatment use among people with SUD. Treatment of all SUD, especially severe SUD, is important given its multiple negative impacts on individual, family, and population health in psychological, cognitive, physical, and social dimensions. Many treatment non-seekers who do not perceive treatment needs will require creative strategies. Our findings show the importance of the two-pronged approach of helping people with SUD increase problem self-perception and assisting them to access treatment.

## Figures and Tables

**Table 1:** Past-year substance use disorder and substance use treatment receipt among USA adults age 18+ (N = 92,233).

		Sample N	Population N (95% CI) in Millions	% (95% CI)
Adults with alcohol or drug use disorder		19,555	46.4 (45.0–47.7)	18.05 (17.63–18.46) ^a^
Of adults with alcohol/drug use disorder				
	Both alcohol and drug use disorder	3586		16.14 (15.27–17.04) ^b^
	Alcohol use disorder, no drug use disorder	8166		45.29 (43–91–46.68) ^b^
	Drug use disorder, no alcohol use disorder	7803		38.57 (37.47–39.69) ^b^
Substance use treatment among adults with alcohol/drug use disorder				
	Received treatment	2766	6.7 (6.2–7.2)	14.37 (13.45–15.33) ^b^
	Did not receive specialty treatment	16,789	39.7 (38.5–40.9)	85.63 (84.67–86.55) ^b^
Among those who did not receive treatment and provided the reasons for treatment non-receipt		16,416		
	Did not seek treatment	15,540	36.7 (35.7–37.7)	94.55 (93.84–95.19) ^c^
	Sought or thought to receive treatment but did not receive	876	2.1 (1.8–2.4)	5.45 (4.81–6.17) ^c^

CI = Confidence intervals. Note: ^a^% (95% CI) of all adult population; ^b^% (95% CI) of adults with alcohol/drug use disorder; ^c^% (95% CI) of those who did not receive treatment and provided reasons for it.

**Table 2:** Characteristics of SUD treatment users versus non-users and of treatment seekers versus non-seekers.

		SUD Treatment Among Those with AUD/DUD			Among Treatment Non-Users		
		Received N = 2766 (14.37%)	Did Not Receive N = 16,789 (85.63%)	*p*	Treatment Seekers N = 876 (5.45%)	Treatment Non-Seeker N = 15,540 (94.55%)	*p*
Age group (%)				0.002			<0.001
	18–25	16.68	20.77		18.04	21.15	
	26–34	20.36	23.39		35.87	22.72	
	35–49	32.79	26.59		24.11	26.67	
	50–64	23.08	19.79		18.19	19.82	
	65+	7.08	9.46		3.79	9.64	
Female (%)		43.81	41.77	0.115	47.20	41.41	0.024
Race/ethnicity (%)				0.195			0.386
	Non-Hispanic White	66.53	62.28		62.98	62.32	
	Non-Hispanic Black	9.95	13.06		10.70	13.18	
	Hispanic	16.50	17.27		16.73	17.27	
	Asian/Pacific Islander	3.23	3.88		3.86	3.82	
	American Indian/Alaska Native	0.91	0.71		0.99	0.70	
	Multi-racial	2.89	2.80		4.74	2.71	
Marital status (%)				<0.001			0.154
	Married	21.98	35.52		29.72	35.90	
	Widowed	4.39	2.81		1.62	2.85	
	Divorced/separated	24.93	14.63		15.04	14.45	
	Never married	48.71	47.04		53.61	46.79	
Residential area (%)				<0.001			0.535
	Large metropolitan area	56.0	50.07		58.49	55.87	
	Small metropolitan area	33.02	33.95		32.16	33.17	
	Nonmetropolitan area	10.98	15.98		9.36	10.96	
Education (%)				<0.001			<0.001
	≤High school	47.50	35.46		30.2	44.0	
	Some college/associate degree	36.23	33.07		41.8	38.0	
	College degree	16.28	31.47		28.0	18.0	
Income (%)				<0.001			0.761
	Below poverty	28.17	16.71		18.25	16.63	
	Up to 2× poverty	26.55	18.98		18.97	18.96	
	More than 2× poverty	45.28	64.31		62.78	64.41	
Any health insurance coverage (%)		91.48	88.31	0.001	83.47	88.72	0.019
Availability of workplace help with substance use problems (%)		22.14	32.34	<0.001	31.03	33.02	0.526
Parole or probation state (%)		12.51	2.43	<0.001	3.79	2.41	0.299
No. of chronic medical conditions, M (SE)		0.76 (0.03)	0.59 (0.01)	<0.001	0.59 (0.05)	0.60 (0.01)	0.807
Types of substance use disorder (%)				<0.001			<0.001
	Both alcohol and drug use disorder	25.05	14.64		35.71	13.5	
	Alcohol, not drug, use disorder	27.0	48.36		42.56	48.73	
	Drug, not alcohol, use disorder	47.95	37.0		21.73	37.76	
Alcohol use disorder (AUD) severity (%)				<0.001			<0.001
	No AUD	47.95	37.0		21.73	37.76	
	Mild	15.37	39.43		16.27	40.92	
	Moderate	10.29	13.69		15.38	13.66	
	Severe	26.39	9.88		46.62	7.65	
Drug use disorder (DUD) severity (%)				<0.001			<0.001
	No DUD	27.0	48.36		42.56	48.73	
	Mild	27.79	31.05		13.19	32.06	
	Moderate	15.78	11.98		10.88	12.02	
	Severe	29.43	8.60		33.37	7.18	
Problem self-perception (%)		60.43	27.87	<0.001	81.71	25.36	<0.001
Past-year mental illness (%)				<0.001			<0.001
	None	32.46	58.21		30.78	60.09	
	Mild	19.73	16.36		21.77	15.84	
	Moderate	18.82	12.26		16.66	11.76	
	Severe	28.99	13.18		30.79	12.32	
Receipt of mental health treatment (%)		74.18	30.80	<0.001	37.05	30.53	0.021
	Inpatient treatment	17.18	1.47	<0.001	1.56	1.37	
	Outpatient treatment	60.84	17.86	<0.001	23.79	17.52	
	Medication therapy	51.24	22.80	<0.001	31.40	22.46	
	Phone/video treatment	46.06	18.44	<0.001	21.50	18.36	

Note: Probability values were calculated based on Pearson’s *χ*^2^ test for categorical variables and *t* test for the number of chronic medical conditions.

**Table 3:** Correlates of treatment receipt (Model 1) and of treatment-seeking (Model 2): Logistic regression results.

		Of Those with Any SUD	Among Treatment Non-Users
		Treatment Users vs. Non-Users aOR (95% CI)	Treatment Seekers vs. Non-Seekers aOR (95% CI)
Age group: vs. 18–25			
	26–34	1.41 (1.13–1.76) **	2.04 (1.48–2.82) ***
	35–49	2.09 (1.65–2.65) ***	1.38 (0.91–2.10)
	50–64	2.09 (1.55–2.82) ***	1.94 (1.07–3.54) *
	65+	1.87 (1.12–3.10) *	1.27 (0.52–3.10)
Male vs. Female		1.18 (1.01–1.39) *	0.67 (0.51–0.88) **
Race/ethnicity: vs. Non-Hispanic White			
	Non-Hispanic Black	0.86 (0.68–1.07)	0.95 (0.58–1.57)
	Hispanic	1.01 (0.76–1.35)	1.08 (0.73–1.60)
	Asian/Pacific Islander	1.23 (0.54–2.77)	1.00 (0.40–2.51)
	American Indian/Alaska Native	0.95 (0.42–2.12)	1.17 (0.52–2.48)
	Multi-racial	0.88 (0.61–1.26)	1.73 (0.97–3.09)
Marital status: vs. Married			
	Widowed	1.75 (1.03–2.97) *	0.53 (0.17–1.63)
	Divorced/separated	1.63 (1.26–2.10) ***	0.91 (0.58–1.45)
	Never married	1.36 (1.06–1.74) *	1.00 (0.75–1.32)
Residential area: vs. Large metropolitan area			
	Small metropolitan area	0.92 (0.77–1.11)	0.82 (0.60–1.14)
	Nonmetropolitan area	1.12 (0.87–1.44)	0.74 (0.50–1.09)
Education: vs. ≤High school			
	Some college/associate degree	0.80 (0.66–0.97) *	1.23 (0.86–1.78)
	College degree	0.48 (0.37–0.63) ***	1.12 (0.76–1.65)
Income: vs. Below poverty			
	Up to 2× poverty	0.87 (0.69–1.10)	0.73 (0.46–1.17)
	More than 2× poverty	0.61 (0.48–0.78) ***	0.77 (0.52–1.13)
Any health insurance coverage		1.46 (1.11–1.93) **	0.71 (0.48–1.04)
Availability of workplace help with substance use problems		0.82 (0.65–1.04)	0.98 (0.73–1.30)
Parole or probation state		3.31 (2.23–4.92) ***	1.01 (0.37–2.72)
No. of chronic medical conditions, M (SE)		0.97 (0.88–1.06)	1.04 (0.88–1.22)
Types of substance use disorder: vs. Alcohol, not drug, use disorder			
	Drug, not alcohol, use disorder	1.87 (1.39–2.52) ***	0.62 (0.38–1.02)
	Both alcohol and drug use disorder	1.17 (0.84–1.63)	0.72 (0.45–1.14)
Alcohol use disorder (AUD) severity: vs. No AUD or mild AUD			
	Moderate	1.63 (1.18–2.25) **	1.95 (1.21–3.11) **
	Severe	3.85 (2.82–5.26) ***	7.07 (4.72–10.57) ***
Drug use disorder (DUD) severity: vs. No DUD or mild severity			
	Moderate	1.54 (1.20–1.97) **	1.79 (1.09–2.96) *
	Severe	2.81 (2.27–3.47) ***	6.85 (4.30–10.91) ***
Problem self-perception		2.12 (1.74–2.58) ***	6.47 (4.59–9.10) ***
Past-year mental illness: No mental illness			
	Mild	1.17 (0.91–1.50)	1.86 (1.33–2.62) **
	Moderate	1.08 (0.83–1.39)	1.83 (1.17–2.86) **
	Severe	1.06 (0.82–1.38)	2.14 (1.39–3.29) **
Receipt of mental health treatment		6.07 (4.73–7.78) ***	0.95 (0.69–1.31)
Overall model statistics		N = 19,548; Population N = 46.4 million; Design df = 50; F (34,17) = 45.23; *p* < 0.001	N = 16,410; Population N = 38.8 million; Design df = 50; F (34,17) = 16.73; *p* < 0.001

* *p* < 0.05; ** *p* < 0.01; *** *p* < 0.001.

**Table 4:** Reasons for treatment non-use among treatment seekers.

Sample N	Reason—Personal	%	Sample N	Reason—Environmental/Structural	%
864	Thought could be handled by oneself	76.34	861	The cost was too high	47.96
850	Not ready to start treatment	61.15	870	Did not know where to go	44.80
852	Not ready to stop/cut use	53.95	852	Not enough time for treatment	41.62
870	Worried about what people would think/say	43.51	820	No health insurance	38.49
857	Worried that the information would not be kept private	34.50	774	Health insurance did not cover	36.97
846	Did not think treatment would help	27.44	856	Thought would lose job/home/child	36.61
858	Afraid to be forced against their will	24.21	838	Could not find a preferred provider	32.86
855	No one would care if I got better	19.34	825	No opening with preferred provider	13.47
856	Family/friends/religion would not like	17.10			

Note: Response categories were not mutually exclusive.

## Data Availability

This study is based on de-identified public-domain data (The National Survey on Drug Use and Health).
